# Supplementary material for: Crawling and Gliding: A Computational Model for Shape-Driven Cell Migration
Source: PLoS Comput Biol. 2015 Oct 21;11(10):e1004280. doi: 10.1371/journal.pcbi.1004280 (PMC4619082; doi:10.1371/journal.pcbi.1004280)
Supplement: S1 Code — (ZIP) [file pcbi.1004280.s012.zip › release/tst/doc/html/ca_8cpp.html]

Tissue Simulation Toolkit: ca.cpp File Reference


Tissue Simulation Toolkit
 0.1.4.1


- Main Page
- Namespaces
- Classes
- Files

- File List
- File Members

Macros |
Functions |
Variables

ca.cpp File Reference

`#include <stdio.h>`  
`#include <math.h>`  
`#include <cstdlib>`  
`#include <cstring>`  
`#include "sticky.h"`  
`#include "random.h"`  
`#include "ca.h"`  
`#include "parameter.h"`  
`#include "dish.h"`  
`#include "sqr.h"`  
`#include "crash.h"`  
`#include "hull.h"`  
`#include "init.xpm"`  
`#include <fstream>`

Include dependency graph for ca.cpp:

|  |  |
| --- | --- |
| Macros | |
| #define | ZYGFILE(Z)   <Z.xpm> |
|  | |
| #define | XPM(Z)   Z ## \_xpm |
|  | |
| #define | ZYGXPM(Z)   XPM(Z) |
|  | |
| #define | ZYGOTE   init |
|  | |

|  |  |
| --- | --- |
| Functions | |
| double | sat (double x) |
|  | |

|  |  |
| --- | --- |
| Variables | |
| double | copyprob [BOLTZMANN] |
|  | |
| Parameter | par |
|  | |

## Macro Definition Documentation

|  |  |  |  |  |  |
| --- | --- | --- | --- | --- | --- |
| #define XPM | ( |  | Z | ) | Z ## \_xpm |

|  |  |  |  |  |  |
| --- | --- | --- | --- | --- | --- |
| #define ZYGFILE | ( |  | Z | ) | <Z.xpm> |

#define ZYGOTE   init

Referenced by CellularPotts::ReadZygotePicture().

|  |  |  |  |  |  |
| --- | --- | --- | --- | --- | --- |
| #define ZYGXPM | ( |  | Z | ) | XPM(Z) |

Referenced by CellularPotts::ReadZygotePicture().

## Function Documentation

|  |  |  |  |  |  |
| --- | --- | --- | --- | --- | --- |
| double sat | ( | double | *x* | ) |  |

References Parameter::saturation.

## Variable Documentation

double copyprob[BOLTZMANN]

Parameter par

Referenced by QtGraphics::TimeStepWrap().


---

Generated on Thu Aug 14 2014 22:04:01 for Tissue Simulation Toolkit by  

 1.8.6
